# Targeting Persistent Neuroinflammation after Hypoxic-Ischemic Encephalopathy—Is Exendin-4 the Answer?

**DOI:** 10.3390/ijms231710191

**Published:** 2022-09-05

**Authors:** Kelly Q. Zhou, Simerdeep K. Dhillon, Laura Bennet, Alistair J. Gunn, Joanne O. Davidson

**Affiliations:** Department of Physiology, The University of Auckland, Auckland 1142, New Zealand

**Keywords:** hypoxic, ischemic, encephalopathy, HIE, Exendin-4, exenatide, anti-inflammatory, neuroinflammation

## Abstract

Hypoxic-ischemic encephalopathy is brain injury resulting from the loss of oxygen and blood supply around the time of birth. It is associated with a high risk of death or disability. The only approved treatment is therapeutic hypothermia. Therapeutic hypothermia has consistently been shown to significantly reduce the risk of death and disability in infants with hypoxic-ischemic encephalopathy. However, approximately 29% of infants treated with therapeutic hypothermia still develop disability. Recent preclinical and clinical studies have shown that there is still persistent neuroinflammation even after treating with therapeutic hypothermia, which may contribute to the deficits seen in infants despite treatment. This suggests that potentially targeting this persistent neuroinflammation would have an additive benefit in addition to therapeutic hypothermia. A potential additive treatment is Exendin-4, which is a glucagon-like peptide 1 receptor agonist. Preclinical data from various in vitro and in vivo disease models have shown that Exendin-4 has anti-inflammatory, mitochondrial protective, anti-apoptotic, anti-oxidative and neurotrophic effects. Although preclinical studies of the effect of Exendin-4 in perinatal hypoxic-ischemic brain injury are limited, a seminal study in neonatal mice showed that Exendin-4 had promising neuroprotective effects. Further studies on Exendin-4 neuroprotection for perinatal hypoxic-ischemic brain injury, including in large animal translational models are warranted to better understand its safety, window of opportunity and effectiveness as an adjunct with therapeutic hypothermia.

## 1. Introduction

Moderate to severe hypoxic ischemic-encephalopathy (HIE) affects 1–3 in 1000 live term born infants in high income countries and is associated with a high risk of death or lifelong disability [1]. Therapeutic hypothermia is the only established treatment and is currently standard care for infants in high-income countries [1,2,3]. Although therapeutic hypothermia significantly improves survival and disability for term infants with moderate to severe HIE, into middle childhood [1,2,3], a recent trial showed that 29% of infants with HIE died or developed moderate to severe disability despite treatment with therapeutic hypothermia [4]. Furthermore, even when the incidence of severe disability is reduced, there are some aspects of neurodevelopment that are still affected despite treatment with hypothermia. For example, studies have shown that children aged 6–8 years, who were treated with therapeutic hypothermia for HIE and did not develop cerebral palsy, performed worse in motor and cognitive tests and had disrupted brain connectivity compared with control children [5,6,7]. 

Additionally, the recent hypothermia for moderate or severe neonatal encephalopathy in low-middle income countries (HELIX) trial showed that therapeutic hypothermia did not reduce the combined outcome of death and disability, but increased the risk of death [8]. However, there are currently no other treatment options available to reduce the burden of brain damage in this setting. Given that 96% of 1.15 million cases of HIE in 2010 occurred in low to middle income countries [9], developing safe and effective treatments for infants in these settings is a critical area of research.

There is thus considerable commitment to finding additive treatments to therapeutic hypothermia. Pre-clinical studies have shown that there is persisting neuroinflammation even after treatment with hypothermia [10,11]. For example, in near-term fetal sheep, there was only a partial reduction in microgliosis and “pro-inflammatory” microglia at 7 days after global cerebral ischemia [11]. Targeting this persistent neuroinflammation may provide additive neuroprotection to therapeutic hypothermia. A Food and Drug Administration (FDA)-approved drug for treating diabetes mellitus called Exendin-4 (Ex-4) has been shown to have neuroprotective effects, which are mediated independently of its insulinotropic action, including anti-inflammatory, anti-apoptotic, anti-oxidative and neurotrophic effects. Early post-insult administration of Ex-4 was highly neuroprotective in a postnatal day (P) 7 mouse model of hypoxic-ischemic brain injury, both when used alone or in combination with hypothermia [12]. The focus of this review is on the role of inflammation after perinatal hypoxic-ischemic brain injury, the potential of Ex-4 to modulate this inflammation, and other effects of Ex-4 that could contribute to neuroprotection.

## 2. Evolution of Injury

**Figure 1:**
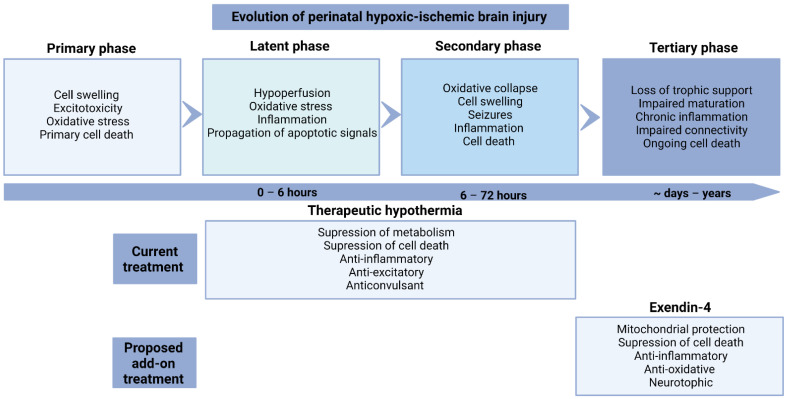
The evolution of perinatal hypoxic-ischemic brain injury and the effects of the current treatment (therapeutic hypothermia) and potential treatment (Exendin-4).

Perinatal hypoxic-ischemic brain injury evolves over time and is classified into four phases (Figure 1) [13]. The primary phase of injury occurs during the hypoxic-ischemic insult, resulting in the failure of oxidative metabolism, anoxic depolarization, cytotoxic edema and necrotic cell death [14]. After the restoration of blood flow and oxygen supply, there is a period of transient recovery of oxidative metabolism, suppressed electroencephalographic (EEG) activity and hypoperfusion [15,16]. After 6 h, this is followed by the secondary phase of “delayed” loss of oxidative metabolism, onset of seizures secondary cell swelling, and increased vascular leakage [17,18,19]. The majority of cell loss after hypoxia-ischemia occurs in this period (6–72 h), through a continuum of necrosis-apoptosis and autophagy [20,21]. The tertiary phase can persist for weeks to years, when there may be persisting low level cell death, due to the loss of trophic support and connectivity problems [22,23]. Long-term impairment may be exacerbated by persistent inflammation, as discussed below. 

## 3. Inflammation after HIE

The inflammatory response after hypoxia-ischemia can be initiated within minutes after the insult, and can persist for weeks to months [24]. Neuroinflammation is likely an important contributor to ongoing deficits. For example, a prospective cohort study of 73 term infants exposed to perinatal asphyxia showed that those who died or were diagnosed with cerebral palsy at a 1 year follow up, had higher levels of interleukin (IL)-1, IL-6 and tumor necrosis factor (TNF) in heel-stick blood samples taken within two days of birth [25]. In the same cohort, at 30 months of age infants who had had higher serum cytokine levels at birth had greater risk of abnormal cognitive and motor outcomes [26]. These effects may persist long term, as shown in infants with cerebral palsy who had higher plasma TNF at 7 years of age, than children in the control group [27].

This inflammatory response involves the resident immune cells in the brain including microglia and astrocytes, and the infiltrating peripheral immune cells such as neutrophils, mast cells and macrophages. These cells release pro-inflammatory factors such as cytokines, chemokines, nitric oxide (NO), reactive oxygen species (ROS), excitatory amino acids and death receptor ligands [28,29,30]. The inflammatory response is crucial for the elimination of cellular debris and facilitating tissue repair following an acute brain insult [24,31,32,33]. However, some of these inflammatory signals can activate deleterious mechanisms that may cause further injury [24,34,35].

### 3.1. Role of Microglia

Microglia are the resident immune cells of the brain and they play an important role in immune surveillance under normal conditions [36]. Resting microglia commonly have a ramified morphology. Aside from their immune function, they are important for normal brain development by mediating apoptosis and pruning of excessive neurons and synapses, and the phagocytosis of debris [37]. In response to brain injury, microglia activate and proliferate as part of the inflammatory response [37]. Activated microglia become amoeboid, with an enlarged cell body and little to no processes. Activated microglia migrate to injury sites and recruit other immune cells, including monocytes and macrophages [37]. 

Activated microglia have various roles, however, the classification of these functions remain controversial [38]. The classical, M1-like polarization is proposed to mediate the progression of inflammation, whereas the alternative or M2-like polarization is suggested to be involved in resolving inflammation [39,40]. M1-like microglia express cluster of differentiation (CD)86, CD16, CD32, produce ROS and nitric oxide synthase (NOS), proteases, a range of interleukins and inflammatory cytokines such as IL-1β, IL-6, and TNF [28,29,30]. The M2-like polarization is associated with expression of CD206 and arginase 1 (Arg-1) and production of anti-inflammatory IL-10 and growth factors, favoring repair [28,29,30]. However, in recent years there has been debate as to whether clear polarization of the microglia phenotypes exists in vivo. It is likely that microglia are much more complex and can simultaneously express both M1 and M2 typical markers [41,42].

In postnatal day (P)7 rats, there was a significant increase in CD45/CD11b positive cells in injured areas assessed at 24 h after transient middle cerebral artery occlusion (MCAO). Interestingly, these cells were predominantly microglia and not infiltrated blood monocytes [31]. Similarly, the number of microglia/macrophage were significantly increased in the hippocampus from 1 day after common carotid artery ligation and hypoxia in P9 mice [43]. The upregulation of microglia may be a biphasic response shown by an initial increase in the number of microglial in the hippocampus at 2 days, followed by a secondary increase in the striatum and cortex at 9 days, after common carotid artery ligation and hypoxia in P9 mice [44]. Similarly, there was a significant upregulation of classically activated CD11b/CD86 positive microglia from whole-brain homogenates at 24 h, followed by a secondary peak at 1 week, which was resolved by 2 weeks to control levels after hypoxia-ischemia in P10 mice [45]. However, alternative microglial activation markers were not assessed in that study. There were significantly increased numbers of ionized calcium-binding adapter molecule 1 (Iba1) positive microglia in the intragyral white matter of the first and second parasagittal gyrus, and the periventricular white matter at one week after global cerebral ischemia in near-term fetal sheep [17,46]. Moreover, Iba1 positive microglial number was still elevated in the same white matter regions as above at 21 days after severe hypoxia-ischemia induced by acute umbilical cord occlusion in preterm fetal sheep [47]. The presence of increased number of microglia weeks after hypoxia-ischemia highlights the chronic nature of inflammation after perinatal hypoxic-ischemic insults.

The functions of activated microglia appear to evolve over time after hypoxia-ischemia. The activation state of microglia and infiltrated macrophages has been characterized in P9 mice after common carotid artery occlusion followed by hypoxia [41]. This study reported an upregulation in messenger ribonucleic acid (mRNA) levels of both pro and anti-inflammatory genes at 24 h after hypoxia-ischemia [41]. Furthermore, the number of classically activated CD86 positive microglia were significantly increased. In contrast, although the absolute numbers of alternatively activated CD206 positive microglia increased, their proportion relative to total numbers of microglia was reduced. Interestingly, there was a population of microglia that did not express either CD86 or CD206, which highlights the complexity of microglial activation polarization [41]. The early rise of pro-inflammatory markers was associated with upregulation of IL-1β and TNF mRNA expression between 1–24 h after hypoxia-ischemia in P7 rats [48]. In another study in P7 rats, both pro-inflammatory (IL-1β, TNF) and anti-inflammatory (IL-10) mRNA expression increased at 3 h after hypoxia-ischemia. At 24 h, TNF expression was still increased compared to sham controls, but less than at 3 h. In addition, anti-inflammatory cytokine TGF-β expression was also upregulated at 24 h, with an increase in CD206 and Iba1 positive microglia [39]. Furthermore, in P7 rats, CD86 positive microglia were predominantly co-localized with IL-1β expression and a small proportion was co-localized with Arg-1, assessed at 6 days after carotid artery ligation and hypoxia [40]. Overall, some studies have indicated that there is a trend towards resolution of inflammation over time. However, as the number of microglia remain upregulated even 3 weeks after asphyxia in preterm fetal sheep [47], further experimental studies are needed to determine the function of microglia at these later time points.

### 3.2. Role of Astrocytes 

Astrocytes are another cell type that can contribute to the inflammatory response after perinatal brain injury. During reactive astrogliosis, astrocytes become hypertrophic and upregulate expression of glial fibrillary acidic protein (GFAP) and potentially form a glial scar around a focal injury [49]. A potential benefit of this response may be to restrict the spread of inflammation and protect neurons and oligodendrocytes [50]. Conversely, the formation of glial scarring may also restrict recovery, partly by inhibiting axonal regeneration [51]. Like microglia, astrocytes can also release pro-inflammatory cytokines, as well as anti-inflammatory mediators [24]. Analogous to the M1/M2 classification of microglia, there is an A1/A2 classification of astrocytes. However, similar to microglia, this classical view may be an oversimplification, as additional activation states are described in vivo [52]. 

Although astrocyte responses are well-characterized in response to ischemic brain injury in the adult brain, the role of reactive astrogliosis in the developing brain is less well understood [24]. Differential astrogliosis responses have been reported between different experimental paradigms of hypoxic-ischemic injury in the developing brain. For example, GFAP expression was increased, despite the number of GFAP positive cells remaining unchanged at 24 h after hypoxia-ischemia in P7 rats [53]. In preterm fetal sheep, there were increased numbers of GFAP positive astrocytes and increased area fraction of GFAP labeling at 3 days after umbilical cord occlusion [54]. By contrast, there were no changes in the number, but a reduction in the area fraction of GFAP positive astrocytes in the parasagittal cortex in near-term fetal sheep at 7 days after carotid artery occlusion [11]. Surprising, there was a differential astrocytic response in the intragyral white matter of the parasagittal cortex in the same study, where there was an increase in both the number astrocytes and area fraction of GFAP labeling, highlighting that the changes in astrocytes are area dependent [11]. Moreover, there was a reduction in area fraction of GFAP positive labeling and the size of astrocytes in neonatal pigs at 3 days after hypoxia [55]. 

## 4. Mechanisms of Therapeutic Hypothermia

The neuroprotective effect of therapeutic hypothermia is mediated through a range of mechanisms beyond merely the suppression of metabolism, including its anti-apoptotic, anti-excitotoxic, anticonvulsant and anti-inflammatory effects, as reviewed in [56]. This section will focus on discussing the anti-inflammatory effects of hypothermia. 

### Anti-Inflammatory Effects

A key mechanism of therapeutic hypothermia is its anti-inflammatory effect. In an in vitro study, hypothermia started before, during and immediately after exposure to hypoxia reduced microglial activation measured by reduced inducible nitric oxide synthase (iNOS) production in both BV-2 microglial cell line and mouse primary microglia cultures [57]. Hypothermia started at 2 h after transient MCAO in adult rats reduced the translocation of a critical transcription factor of inflammation NF-κB [58]. There was also subsequent suppression of proteins regulated by NF-κB, such as iNOS [59], intercellular adhesion molecule-1 [60] and inflammatory cytokines including TNF [58]. Hypothermia started at 90 min after global cerebral ischemia in near-term fetal sheep is consistently associated with at least partially suppressing the number of microglia [10,46,61]. There is recent evidence to suggest that hypothermia may also play a role in modulating the polarization of microglia. Hypothermia started immediately after common carotid artery ligation and hypoxia was associated with suppressing the expression of “pro-inflammatory” CD 86 and increased expression of “anti-inflammatory” CD206 in P9 rats [62]. Recently, it has been reported that hypothermia partially reduced CD86 expression in the cortex, but not in the white matter in near-term fetal sheep at 7 days after global cerebral ischemia [11]. CD206 was also significantly elevated after ischemia, but was not altered by hypothermia. The difference between the rat study and fetal sheep study, may in part be related to the different protocols of hypothermia. It is likely that the loss of efficacy to suppress pro-inflammatory microglia may be related to increasing delay before the start of hypothermia. However, a longer delay before the start of hypothermia is more realistic clinically. 

## 5. Is Chronic Inflammation after Hypothermia Deleterious?

Recent data have shown that numbers of microglia in the parasagittal cortex and in the intragyral white matter of the parasagittal gyrus were both inversely correlated with EEG power at day 7 after global cerebral ischemia in near-term fetal sheep [11]. That is to say, higher numbers of microglial are correlated with worse recovery of EEG activity. However, correlation does not mean causation. Speculatively, this correlation is due to the combination of milder residual neuronal loss seen in hypothermia treated animals, as well as an effect of the persistent microgliosis. Neuroinflammation also has beneficial effects as mentioned above, in terms of clearing of cellular debris following injury and promoting repair. In fact, the depletion of microglia in P10 mice exposed to common carotid artery ligation and hypoxia, was associated with an exacerbation of brain damage [63]. This indicates that the net effect of the microglial response is beneficial. However, at least at the 7 day time point, there still is a predominance of pro-inflammatory microglia present. To investigate whether chronic inflammation does impair recovery from hypoxic-ischemic brain injury, therapeutic agents that can target pro-inflammatory processes, without affecting the beneficial effects of inflammation, i.e., therapies which can immunomodulate “M1” microglia to “M2” microglia should be tested.

## 6. Exendin-4

### 6.1. Background

Ex-4 is a 39 amino acid peptide found in the saliva of the Gila Monster (Heloderma suspectum) [64]. Ex-4 is a glucagon-like peptide 1 (GLP-1) receptor agonist that is structurally similar to the endogenous incretin hormone GLP-1. The therapeutic potential of the synthetic form of Ex-4 (also known as exenatide) was first investigated in the treatment of diabetes, as GLP-1 receptor agonists regulate blood glucose by promoting insulin secretion in the pancreas [65]. Ex-4 has a longer half-life and greater binding affinity to the GLP-1 receptor than GLP-1 [66,67]. Currently, Ex-4 is Food and Drug Administration (FDA) approved for the treatment of type II diabetes. The beneficial neurological effects of Ex-4 were first identified in diabetic patients taking Ex-4 in whom there was an improved sense of well-being and quality of life reported, as well as reduced incidences of depression and anxiety, compared with those taking insulin [68]. There has been immense interest in studying Ex-4 for the treatment of neurodegenerative diseases, stroke and myocardial disease due to its anti-inflammatory, anti-apoptotic and mitochondrial protective properties, discussed below [69,70,71,72,73,74]. There are currently 6 clinical trials registered to determine safety and efficacy of Ex-4 for treatment of Parkinson’s disease, 2 ongoing trails for ischemic stroke and 1 completed trial for Alzheimer’s disease.

### 6.2. Preclinical Studies for HIE

A seminal study of the effect of Ex-4 (4 doses 0.5 µg/kg i.p.) in P7 mice showed that there was a reduction in infarction size, cell death and inflammation associated with Ex-4 treatment started at 2 h after common carotid ligation and hypoxia [12]. When Ex-4 (0.5 µg/kg i.p.) given in conjunction with hypothermia, both started at 10 min after hypoxia-ischemia, this was associated with improved macroscopic tissue score (from 1.5 to 0.5 on a 4-point scale) compared with hypothermia alone in P10 mice [12]. However, overall infarct volume was not significantly different between the combined group and hypothermia only [12]. Neuroprotection with Ex-4 has not yet been investigated in a large animal translational model of perinatal hypoxia-ischemia, either alone as a sole treatment, or in combination with a clinically relevant hypothermia protocol. Moreover, there is a lack of systematic information on the realistic window of opportunity for treatment. As there have been limited studies of the effect of Ex-4 in the setting of perinatal brain injury, the studies discussed in the sections below are predominantly based on models of other diseases.

## 7. Anti-Inflammatory Effects of Exendin-4

**Figure 2:**
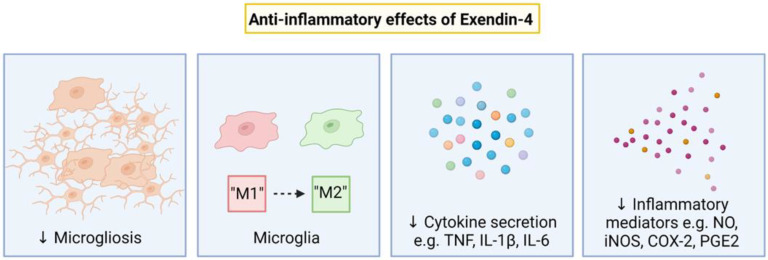
A schematic diagram showing the anti-inflammatory effect of Exendin-4. ↓: decrease.

Ex-4 has been shown to have profound anti-inflammatory effects that may contribute to neuroprotection. Ex-4 administration in neonatal mice born to dams exposed to systemic inflammation induced by lipopolysaccharide (LPS) was associated with increased numbers of IL-10 expressing neutrophils, and suppressed expansion of CD8^+^ regulatory T cells [75]. The number of microglia and iNOS expression by microglia was reduced in adult mice treated with Ex-4 after MCAO [76] (Figure 2). Ex-4 administration was associated with reduced total number of microglia and number of amoeboid microglia in the spinal cord in a rat model of experimental autoimmune encephalomyelitis [77]. Further, Ex-4 treatment (4 doses 0.5 µg/kg i.p.) in P7 mice exposed to common carotid ligation and hypoxia, was associated with a reduction “microglial activation” marker alphaM, αmβ2 labeling [12]. 

Ex-4 has also been shown to modulate microglial polarization to be more “anti-inflammatory” [77,78,79]. For example, Ex-4 (30 µg/kg i.p. or intra-amniotically to the dam) induced anti-inflammatory macrophage polarization (CD11b^+^ F4/80^+^ IL-10^+^ cells) in lung and large intestine tissues in neonatal mice born to dams exposed to systemic inflammation induced by LPS [75]. In human monocytes exposed to LPS in vitro, Ex-4 (10 nM) increased the expression of “M2” markers IL-10 and Arg-1, but decreased the expression of “M1” markers TNF and IL-1β and iNOS [80]. Ex-4 administration (50 µg/kg i.p. at 1.5 h, followed by 0.2 µg/kg daily) was associated with an upregulation in the expression of “M2” markers CD206 but not Arg-1 and YM1/2 after transient MCAO in adult mice [78]. However, there were no significant differences in the expression of CD86 and iNOS between the Ex-4 and vehicle groups. Ex-4 treatment (5 μg/kg, i.p. daily) suppressed pro-inflammatory genes IL-1β, IL-6, IL-17 and TNF in rats with experimental autoimmune encephalomyelitis [77]. This was likely mediated by the suppression of the NF-κB pathway in activated microglia [77]. Further, Ex-4 (10 nM) promoted the expression of “M2” genes Arg-1, CD206 and IL-4 in primary rat microglia, and this was attenuated by the GLP-1 receptor antagonist exendin(9–39) [79].

### Inflammatory Pathways Targetted by Exendin-4

Pre-clinical studies have shown that Ex-4 can modulate inflammation through many different inflammatory pathways. For example, in peripheral blood mononuclear cells collected from patients with diabetes, treatment with Ex-4 reduced the secretion of pro-inflammatory cytokines TNF, IL-1β and IL-6, mediated through the suppression of the mitogen-activated protein kinase (MAPK) signaling pathway [81]. In an adult rat model of stroke, Ex-4 treatment (1 nM, i.c.v.) administered 30 min before by transient middle cerebral artery occlusion was associated with inhibiting inflammatory COX-2 expression, through the suppression of phosphor-JNK [82]. Conversely in the group that was administered a GLP1-R antagonist exendin(9–39) (1 nM, i.c.v.) in the same study, this anti-inflammatory effect was abolished [82]. Ex-4 pretreatment (10 or 20 nM) in murine macrophage RAW 264.7 cells exposed to LPS inhibited the expression of inflammatory mediators including iNOS, COX-2, PGE2 and NO and pro-inflammatory cytokines TNF, IL-1β and IL-6 [83]. The reduction in these inflammatory responses were mediated by the suppression of the JNK and AP-1 pathway and the NF-κB pathway [83]. 

## 8. Other Effects of Exendin-4

### 8.1. Mitochondrial Protection

Ex-4 has mitochondrial protective effects that likely contribute to neuroprotection. Ex-4 improved mitochondrial function, integrity and respiratory control rate in rodent models of Alzheimer’s disease [72,84]. There was preserved membrane potential and decreased Cyt c release after Ex-4 treatment (10 µg, i.p.) given immediately after spinal cord injury in rats [85]. A study showed pre-treatment with Ex-4 (100 nmol/L) in a cytokine-stimulated pancreatic beta cell line INS-1 reduced apoptosis, damage to electron transport chain proteins, and subsequent ROS production [86]. Further, Ex-4 pretreatment (100 nmol/L) protected microglia by reducing mitochondrial potential loss, ROS concentration, Cyt c and Smac release in mouse pancreatic β-cell tumor Min6 cells exposed to oxidative stress [87]. These factors of mitochondrial protection likely contribute to the anti-apoptotic effects of Ex-4.

### 8.2. Anti-Apoptotic

Ex-4 has consistently reduced apoptotic cell death in models of perinatal hypoxic-ischemic brain injury, stroke and spinal cord injury [12,69,76,85]. In P7 mice treated with Ex-4 after common carotid artery ligation and hypoxia, there was a reduction in numbers of terminal deoxynucleotidyl transferase dUTP nick end labeling (TUNEL) positive cells, indicating reduced cell death [12]. Further, there was a reduction in the number of TUNEL positive dying cells, and an increase in intracellular cyclic adenosine monophosphate (cAMP) in the Ex-4 treatment group (10 µg i.v.) in adult mice given immediately after MCAO [76]. Activation of the GLP-1 receptor is known to stimulate adenylyl cyclase, resulting in the increase in cAMP levels [76]. This in turn can activate the cAMP response element-binding protein signaling pathways that has neuroprotective effects, including the upregulation of anti-apoptotic Bcl-2 [88]. Additionally, hippocampal neuronal survival was improved and an increase in the Bcl-2/Bax ratio was seen after Ex-4 administration (1 µg/kg, i.p.), started before bilateral carotid artery occlusion in adult gerbils [69].

### 8.3. Anti-Oxidation

Another mechanism contributing to the neuroprotective effect of Ex-4 may be its anti-oxidative effects. For example, Ex-4 (10 µg, i.v.) reduced oxidative stress, shown by a reduction in the number of 8-Hydroxy deoxyguanosine (oxidative DNA damage product) and 4-hydroxy 2-hexenal (lipid peroxidation product) positive cells in adult mice after MCAO [76]. Additionally, there was reduced lipid peroxidation product malodialdehyde and increased free radical scavenger glutathione levels in the brain associated with Ex-4 administration (10 µg, i.p.) in rats with spinal cord injury, showing an anti-oxidative effect [85]. Ex-4 treatment (50 nmol/L) also attenuated the production of superoxide ions in peripheral blood mononuclear cell cultures isolated from patients with diabetes [81].

### 8.4. Neurotrophic

Ex-4 has also been shown to have neurotrophic effects. For example, Ex-4 administration (0.1 μg/kg, twice daily, s.c.) to diabetic mice was associated with an increase in the expression of neurotrophin BDNF and CREB a transcription factor regulating BDNF expression in the hippocampus [89]. In turn, the increase in BDNF and CREB were associated with improved performance in the modified elevated plus maze and passive avoidance tests, showing improved memory function, compared with the vehicle group [89]. In adult rat dorsal root ganglion neurons in culture, there was a dose dependent response (1–100 nM) of Ex-4 on promoting neurite outgrowth [90]. In vivo, Ex-4 administration (1 μg/kg i.p. twice daily) promoted cell proliferation and neuroblast differentiation in the subgranular zone of the dentate gyrus, compared to the vehicle group [91]. 

## 9. Timing of Exendin-4 Administration

We have discussed the broad range of effects of Ex-4 and its potential for treating perinatal hypoxic-ischemic brain injury. The next pragmatic question is when is the most beneficial time to administer Ex-4? In the Rocha-Ferreira study, Ex-4 was administered at the same time as the start of hypothermia (10 min after hypoxia-ischemia), which was associated with an improved macroscopic tissue score compared with hypothermia alone. Therefore, this study suggests that concurrent treatment is beneficial. However, given there is persistent inflammation following therapeutic hypothermia [11], this raises the possibility of modulating this inflammation after the end of hypothermia (Figure 1). Future studies are needed to investigate the most optimal timing of Ex-4 administration.

## 10. Safety of Exendin-4 in Infants

Ex-4 is currently only approved for use in adults for treating type II diabetes. Therefore, if sufficient pre-clinical data show that Ex-4 is neuroprotective, the safety profile of Ex-4 in infants will have to be carefully established. Ex-4 treatment did not alter blood glucose levels in neonatal mice, compared with control animals [12]. This is reassuring given that Ex-4 reduces blood sugar and that hypoglycemia is associated with adverse outcomes in babies with HIE undergoing hypothermia [92]. Future studies conducted in large animal models should also carefully monitor physiological and biochemical parameters, including heart rate and arterial blood pressure to assess whether there are any adverse effects. 

## 11. Other GLP-1R Agonists

There are other FDA approved GLP-1R agonists available, which are gaining increasing interest for neuroprotection. Liraglutide is a modified form of the human GLP-1 protein, sharing 97% homology with GLP-1, but with an extended elimination half-life compared with GLP-1 [93]. To our knowledge, there has only been preclinical study that has investigated the effect of Liraglutide for the treatment of perinatal hypoxia-ischemia. In this study, P7 rats injected with Liraglutide (200 nmol/kg/day) starting immediately after common carotid artery ligation and hypoxia, had reduced infarct volume, reduced cerebral edema and reduced expression of pro-inflammatory cytokines [94]. However, the effect of delayed administration of Liraglutide, which is more clinically relevant, has not yet been investigated. Semaglutide is a modified version of Liraglutide that has a longer half-life allowing for the potential of single dosing rather than multiple doses or a continuous infusion [95]. However, the neuroprotective effect of Semaglutide has yet to be investigated in a perinatal model of hypoxia-ischemia. 

## 12. Conclusions

There is now considerable evidence of prolonged neuroinflammation after hypoxia-ischemia. This continuing inflammation may, at least in part, contribute to the functional and histological deficits seen clinically and experimentally even after treatment with hypothermia. This suggests that neuroinflammation may be an important target to further improve treatment of HIE. Ex-4 is a promising drug that has potent anti-inflammatory effects and, after early intervention, has shown neuroprotective effects in neonatal mice exposed to HI [12]. Excitingly, there was an additive neuroprotective effect when administered with hypothermia in neonatal mice exposed to HI [12]. Further studies on the effects of Ex-4 and other GLP-1R agonists are now needed in large translation animal models of perinatal hypoxia-ischemia, to examine its safety, window of opportunity for benefit and effectiveness in different settings.
